# Endogenous n-3 Polyunsaturated Fatty Acids Attenuate T Cell-Mediated Hepatitis *via* Autophagy Activation

**DOI:** 10.3389/fimmu.2016.00350

**Published:** 2016-09-13

**Authors:** Yanli Li, Yuan Tang, Shoujie Wang, Jing Zhou, Jia Zhou, Xiao Lu, Xiaochun Bai, Xiang-Yang Wang, Zhengliang Chen, Daming Zuo

**Affiliations:** ^1^Department of Immunology, School of Basic Medical Sciences, Southern Medical University, Guangzhou, China; ^2^Department of Cell Biology, School of Basic Medical Sciences, Southern Medical University, Guangzhou, China; ^3^Department of Human Molecular Genetics, Virginia Commonwealth University, Richmond, VA, USA; ^4^Guangdong Province Key Laboratory of Proteomics, Southern Medical University, Guangzhou, China; ^5^State Key Laboratory of Organ Failure Research, Guangdong Provincial Key Laboratory of Viral Hepatitis Research, Department of Infectious Diseases, Nanfang Hospital, Southern Medical University, Guangzhou, China

**Keywords:** omega-3 polyunsaturated fatty acids, immune-mediated liver injury, concanavalin A-induced hepatitis, T cell activation, autophagy

## Abstract

Omega-3 polyunsaturated fatty acids (n-3 PUFAs) exert anti-inflammatory effects in several liver disorders, including cirrhosis, acute liver failure, and fatty liver disease. To date, little is known about their role in immune-mediated liver diseases. In this study, we used *fat-1* transgenic mice rich in endogenous n-3 PUFAs to examine the role of n-3 PUFAs in immune-mediated liver injury. Concanavalin A (Con A) was administered intravenously to wild-type (WT) and *fat-1* transgenic mice to induce T cell-mediated hepatitis. Reduced liver damage was shown in Con A-administrated *fat-1* transgenic mice, as evidenced by decreased mortality, attenuated hepatic necrosis, lessened serum alanine aminotransferase activity, and inhibited production of pro-inflammatory cytokines (e.g., TNF-α, IL-6, IL-17A, and IFN-γ). *In vivo* and *in vitro* studies demonstrated that n-3 PUFAs significantly inhibited the activation of hepatic T cells and the differentiation of Th1 cells after Con A challenge. Further studies showed that n-3 PUFAs markedly increased autophagy level in Con A-treated *fat-1* T cells compared with the WT counterparts. Blocking hepatic autophagy activity with chloroquine diminished the differences in T cell activation and liver injury between Con A-injected WT and *fat-1* transgenic mice. We conclude that n-3 PUFAs limit Con A-induced hepatitis *via* an autophagy-dependent mechanism and could be exploited as a new therapeutic approach for autoimmune hepatitis.

## Introduction

Autoimmune hepatitis is a medical problem in which the body’s immune system attacks the liver, causing cirrhosis and liver failure (1). Although the etiology is not entirely understood, impaired apoptosis and excessive T cell activation are believed to be associated with the pathophysiology of autoimmune hepatitis (2, 3). The T cell responses involved in autoimmune hepatitis comprise the predominant IFN-γ-producing Th1 cells over IL-4-producing Th2 cells and the reduced frequency and function of regulatory T cells (Tregs) (4–7). It is of critical importance to identify new immune modulators that can counter-regulate the hepatic immune response, especially the T cell response, in autoimmune hepatitis.

Omega-3 polyunsaturated fatty acids (n-3 PUFAs) are a collection of polyunsaturated fatty acids, including eicosapentaenoic acid (EPA) and docosahexaenoic acid (DHA) from fish and alpha-linolenic acid (ALA) in plants (8), which have acknowledged effects on both structural integrity and function of cellular membranes. Emerging evidence showed that n-3 PUFAs could regulate the inflammatory response by decreasing inflammatory cytokines and reactive oxygen production (9). *Fat-1* transgenic mice, which express the *Caenorhabditis elegans fat-1* gene, are capable of synthesizing n-3 PUFAs from the n-6 type, leading to elevated amounts of n-3 PUFAs in their tissues compared with the wild-type (WT) littermates (10). Thus, these mice exhibit more anti-inflammatory derivatives generated from n-3 PUFAs (e.g., resolvin E1, resolvin D3, protectin D1, and maresin 1), resulting in protection against inflammatory disorders in different organs, such as allergic airway inflammation, chemically induced colitis, pancreatitis, and diabetic neuropathy (11–14). Since *fat-1* transgenic mice have significant endogenous amounts of n-3 PUFAs in their liver tissue (15), the function of n-3 PUFAs in liver injury and inflammation has been investigated. *Fat-1* transgenic mice developed less severe d-galactosamine/lipopolysaccharide (d-GalN/LPS)-induced inflammatory liver injury than WT mice, associated with a reduction of pro-inflammatory cytokines (e.g., TNF-α, IL-1β, IL-6, and IFN-γ) (15). Moreover, tissue n-3 PUFAs protected against acute ethanol-induced hepatic steatosis and diet-induced fatty liver disease in *fat-1* transgenic mice, through activation of cholesterol catabolism to bile acid and downregulation of hepatic inflammatory response (16, 17). However, the effect of endogenous n-3 PUFAs on liver immune responses that involve hepatic T lymphocytes remains unclear.

Intravenous injection of mice with T cell mitogen concanavalin A (ConA) induces polyclonal activation of T lymphocytes, resulting in a liver-specific inflammatory response (18). This model is characterized by elevated serum levels of alanine transaminase (ALT) and pro-inflammatory cytokines (e.g., TNF-α, IL-6, and IFN-γ), as well as infiltration of T lymphocytes and necrosis of hepatocytes in the liver tissue (4, 19). Therefore, Con A-induced hepatitis is a well-established murine model that can simulate the pathophysiology of human autoimmune hepatitis and has been extensively employed to elucidate the underlying mechanisms of T cell-mediated autoimmune hepatitis. In this study, we used *fat-1* transgenic mice to explore the protective effect of endogenous n-3 PUFAs on liver injury in the model of autoimmune hepatitis induced by Con A. The results showed that *fat-1* transgenic mice were resistant to Con A-induced hepatitis, which attributed to the suppressed T cell activation and Th1 differentiation in the presence of n-3 PUFAs. We also provided evidences that endogenous n-3 PUFAs enhanced T cell autophagy upon Con A challenge, which may be involved in the inhibition of T cell activation and subsequent liver injury. In summary, our findings revealed that hepatic n-3 PUFAs controlled T cell responses during immune-mediated hepatitis, which may be potentially employed as a new therapeutic strategy for autoimmune hepatitis.

## Materials and Methods

### Mice

Wild-type C57BL/6 mice were purchased from the Laboratory Animal Center of Southern Medical University (Guangzhou, China). *Fat-1* transgenic mice were backcrossed with WT C57BL/6 mice, and the *fat-1* genotypes of each animal were characterized using isolated genomic DNA from mouse tails by PCR analysis as we previously described (20). All animal experiments in this study were approved by the Welfare and Ethical Committee for Experimental Animal Care of Southern Medical University.

### Reagents

Con A, chloroquine, and DHA were purchased from Sigma–Aldrich (St. Louis, MO, USA). Antibodies were obtained from Cell Signaling Technology (Danvers, MA, USA), including the antibodies against p62 (Cat# 5114), LC3 (D11), phospho-STAT1 (58D6), STAT1 (D1K9Y), phospho-STAT3 (D3A7), STAT3 (D3Z2G), phospho-NF-κB p65 (93H1), NF-κB p65 (C22B4), and GAPDH (D16H11). Mouse monoclonal antibodies against CD3 (145-2C11), CD4 (RM4-5), IFN-γ (XMG1.2), and CD69 (H1.2F3) were purchased from BD Pharmingen (San Jose, CA, USA). Anti-NK1.1 (PK136), anti-CD16/CD32 (2.4G2), goat anti-rabbit IgG, and 7-aminoactinomycin D (7-AAD) were from MultiSciences (Hangzhou, China). Also, 5-(and-6)-carboxyfluorescein diacetate succinimidyl ester (CFSE) was purchased from Invitrogen (San Diego, CA, USA).

### Con A-Induced Hepatitis Model

Con A-induced hepatitis was initiated as described previously with modification (21). Briefly, Con A (C2010, Sigma–Aldrich) was dissolved in PBS at 1 mg/ml, and age-matched mice received an intravenous injection of Con A to induce hepatitis. The high dose (35 mg/kg body weight) of Con A was used to generate survival curves in WT and *fat-1* transgenic mice, while the low dose (15 mg/kg body weight) of Con A permitted assessment of the liver pathology with hematoxylin and eosin (H&E) staining and other *in vitro* assays at indicated time after Con A challenge. To test the benefit of dietary n-3 PUFAs in immune-mediated liver injury, WT mice were fed with an n-3 PUFAs-enriched diet, as we previously described (20), for 3 weeks before Con A administration. For autophagy inhibition, chloroquine was injected intraperitoneally at the dose of 40 mg/kg body weight 1 h before Con A administration.

### TUNEL Assay

Formalin-fixed and paraffin-embedded tissue sections of liver were deparaffinized in xylene, then gradually rehydrated in decreasing concentrations of ethanol and distilled water. Subsequently, proteinase K permeabilized sections were subjected to incubation with TdT enzyme and fluorochrome mixture (Promega, Madison, WI, USA) for 1 h at 37°C in the dark. After DAPI (Roche, Mannheim, Germany) staining, the slides were analyzed under a fluorescence microscope.

### Serum ALT Assay and Cytokine Assessment

Individual mouse serum was collected at different time points after Con A injection. Serum alanine aminotransferase (ALT) activity was measured with a commercial kit (Jiancheng Biotech, Nanjing, China), according to the manufacturer’s instruction. Cytokine levels in the sera and cell culture supernatants were assessed using commercial ELISA kits purchased from eBioscience (San Diego, CA, USA).

### Western Blotting Analysis

Protein samples were separated on SDS-polyacrylamide gels and then transferred onto polyvinylidene fluoride (PVDF) membranes (Millipore, Billerica, MA, USA). Bovine serum albumin (BSA, 5%) was used to block non-specific sites of the membranes for at least 1 h at room temperature. Subsequently, the membranes were incubated overnight at 4°C with primary antibodies, followed by incubation with the horseradish peroxidase-conjugated secondary antibody for 1 h at room temperature. Finally, the membranes were washed three times, and detection of the target protein was conducted with enhanced chemiluminescence (Thermo Fisher, Carlsbad, CA, USA).

### Preparation of Liver Mononuclear Cells and Purification of Hepatic T Cells

Mouse livers were removed and pressed through a 200-gage stainless steel mesh. The filtrate containing non-parenchymal cell was centrifuged at 50 × *g* for 5 min. Supernatants containing mononuclear cells (MNCs) were collected, followed by washing once with PBS. The cells were resuspended in 30% Percoll (GE Healthcare, Uppsala, Sweden) and gently overlaid onto 70% Percoll. After centrifugation at 1000 × *g* for 20 min, liver MNCs were collected from the interphase, washed twice with PBS, and then resuspended for further proliferation assay and FACS analysis. T cells were purified from liver MNCs using mouse T lymphocyte enrichment set from BD Biosciences (San Jose, CA, USA).

### Flow Cytometry

Isolated liver MNCs were resuspended in PBS containing 1% BSA. NKT cells and T cells were determined by staining with anti-CD3, anti-CD4, anti-NK1.1, and anti-CD69 (BD Pharmingen). For detection of IFN-γ and LC3, cells with surface staining were fixed and permeabilized by Cytofix/Cytoperm kit (eBioscience), further stained with anti-IFN-γ or anti-LC3 antibodies. To evaluate nuclear factor-κB (NF-κB) activity in T cells, hepatic MNCs from Con A-injected mice were permeabilized by methanol followed stained with fluorescence-conjugated antibodies against phospho-NF-κB p65 and CD3. T cell apoptosis was monitored by FACS analysis using Annexin V/propidium iodide (PI) staining, according to the manufacturer’s instruction (Keygen Biotech, Nanjing, China).

### Isolation of RNA and qRT-PCR Analysis

Mice liver or hepatic MNCs’ total RNA was extracted using Trizol (Invitrogen) and then transcribed into cDNA using the reverse transcription kit (TaKaRa, Dalian, China), as instructed by the manufacturer. SYBR Green quantitative RT-PCR was performed to determine the gene expression level using a 7900HT fast real-time PCR system (Applied Biosystems, San Francisco, CA, USA), according to the protocols provided with the SYBR Premix EX Taq (TaKaRa). The levels of target gene were normalized with respect to GAPDH gene expression. The primer sequences used in the experiment are shown in Table 1.

**Table 1:** **Primers for inflammatory cytokines and T cell-specific transcript factors**.

	Forward primer (5′–3′)	Reverse primer (5′–3′)
IFN-γ	CATCAGCAACAACATAAGCGTCA	CTCCTTTTCCGCTTCCTGA
TNF-α	CTCTTCTGCCTGCTGCACTTTG	ATGGGCTACAGGCTTGTCACTC
IL-6	TACCACTTCACAAGTCGGAGGC	CTGCAAGTGCATCATCGTTGTTC
IL-17A	CAGACTACCTCAACCGTTCCAC	TCCAGCTTTCCCTCCGCATTGA
IL-4	ATCATCGGCATTTTGAACGAGGTC	ACCTTGGAAGCCCTACAGACGA
IL-10	CGGGAAGACAATAACTGCACCC	CGGTTAGCAGTATGTTGTCCAGC
T-bet	CCACCTGTTGTGGTCCAAGTTC	CCACAAACATCCTGTAATGGCTTG
GATA3	CCTCTGGAGGAGGAACGCTAAT	GTTTCGGGTCTGGATGCCTTCT
RORγt	CCGCTGAGAGGGCTTCAC	TGCAGGAGTAGGCCACATTACA
Foxp3	CCTGGTTGTGAGAAGGTCTTCG	TGCTCCAGAGACTGCACCACTT
GAPDH	CATCACTGCCACCCAGAAGACTG	ATGCCAGTGAGCTTCCCGTTCAG

### *In vitro* T Cell Stimulation

Liver MNCs (4 × 10^5^ per well) were treated with various concentration of Con A in the flat-bottomed 96-well plate for 72 h for proliferation assay. Supernatants were collected at 48 h for cytokine assays using ELISA. Purified hepatic T cells were stimulated with 5 μg/ml of Con A for indicated time followed by immunoblotting and FACS analysis. In some studies, DHA was added 4 h before treatment with Con A.

### Statistical Analysis

The experimental data were evaluated by calculating the mean ± SD. One-way ANOVA was used for comparisons among multiple groups. Differences between two groups within experiments were analyzed by Student’s *t-*test. Comparison of the survival curves was performed using the log-rank test. Values of *p* < 0.05 were considered statistically significant.

## Results

### n-3 PUFAs Play a Protective Role in Con A-Induced Fulminant Hepatitis

**Figure 1:**
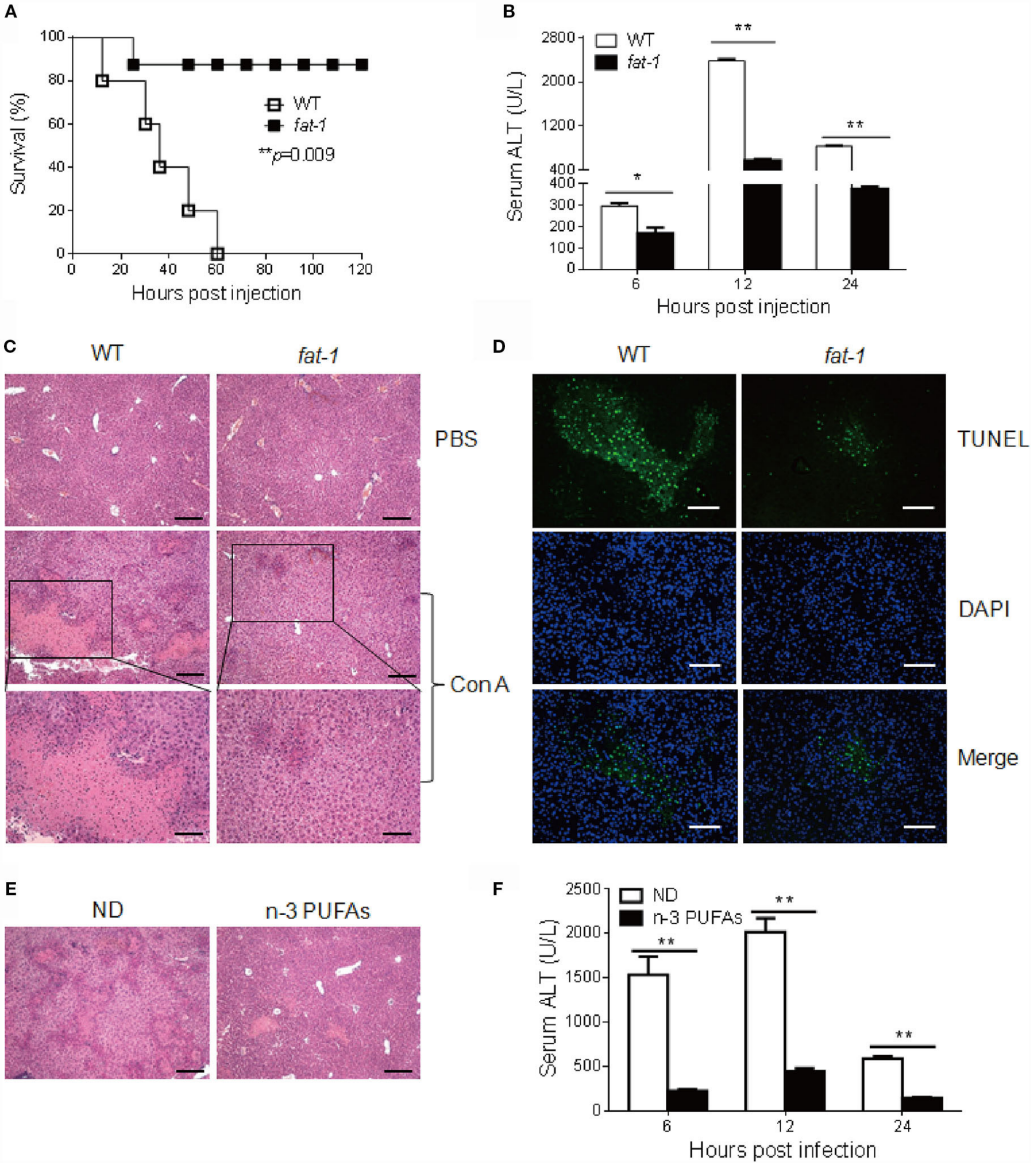
**n-3 PUFAs protect mice from Con A-induced hepatitis**. **(A)** WT and *fat-1* transgenic mice (*n* = 10) were injected with Con A at the dose of 35 mg/kg, and overall survival of mice was monitored (*p* = 0.009). **(B–D)** A low dose of Con A (15 mg/kg) was injected into WT and *fat-1* transgenic mice (*n* = 5). Serum ALT activities in different time points were determined **(B)**. Histological analysis of mouse livers was performed with H&E staining. Scale bars = 100 μm. Bottom panels showed the higher-magnification views of the necrotic area. Scale bars = 50 μm. **(C)**. The apoptosis in mice liver tissues was evaluated by fluorescence TUNEL staining. Scale bars = 50 μm. **(D)**. *p < 0.05, **p < 0.01. Data are representative of three independent experiments with similar results. **(E,F)** WT mice were injected with Con A (15 mg/kg) after consuming normal diet (ND) or n-3 PUFAs-enriched diet (n-3 PUFAs) for 3 weeks (*n* = 5 each). Histology of liver **(E)** and serum ALT **(F)** were examined 24 h following Con A administration. Scale bars = 100 μm. **p < 0.01. Data are representative of two independent experiments with similar results.

To define the role of n-3 PUFAs in Con A-induced hepatitis, sex- and age-matched C57BL/6 WT and *fat-1* transgenic mice were treated with a high dose of Con A (35 mg/kg body weight), and the survival rate of mice was determined. Strikingly, all of the WT mice died within 60 h. In contrast, 90% of *fat-1* transgenic mice survived the challenge (Figure 1A), indicating a protective role of endogenous n-3 PUFAs against Con A-induced liver damage. To evaluate the effect of n-3 PUFAs on liver injury, mice received a low dose of Con A (15 mg/kg body weight), followed by the examination of serum ALT activity and liver pathological changes. Sera collected from *fat-1* transgenic mice showed low levels of ALT activity compared to that from WT mice after Con A injection (Figure 1B). Liver histological changes were examined at 16 h after Con A challenge. The result showed that liver tissues from *fat-1* transgenic mice displayed less hepatocyte necrosis and disseminated hemorrhage than those from WT mice (Figure 1C). Besides, the TUNEL positive cells were more abundant in the livers of Con A-treated WT mice than those from *fat-1* transgenic mice, indicating endogenous n-3 PUFAs could suppress the severe necrosis and apoptosis in the liver (Figure 1D). To further verify the protective effect of n-3 PUFAs on Con A-induced hepatitis, WT mice were fed with n-3 PUFAs-enriched diet for 3 weeks prior to Con A administration. In contrast to mice with normal diet (ND), mice receiving n-3 PUFAs-enriched diet showed minor liver damage after Con A injection as reflected by hepatic pathology (Figure 1E) and serum ALT levels (Figure 1F).

**Figure 2:**
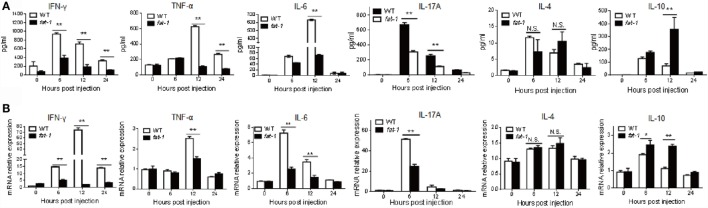
**Endogenous n-3 PUFAs differentially regulate the expression of cytokines during Con A-induced liver injury**. **(A)** Serum levels of IFN-γ, TNF-α, IL-6, IL-17A, IL-4, and IL-10 were determined at 0, 6, 12, and 24 h post-Con A administration (*n* = 5). **(B)** Relative intrahepatic mRNA expression levels of IFN-γ, TNF-α, IL-6, IL-17A, IL-4, and IL-10 were measured by quantitative RT-PCR analysis at 0, 6, 12, and 24 h post-Con A administration and expressed as a ratio to GAPDH (*n* = 5). *p < 0.05, **p < 0.01. Data shown represent three independent experiments with similar results.

Next, we compared the serum levels of cytokines between Con A-treated WT and *fat-1* transgenic mice to determine the protective role of endogenous n-3 PUFAs in Con A-induced hepatitis. Attenuated liver damage in *fat-1* transgenic mice was accompanied by a pronounced reduction of pro-inflammatory cytokines (i.e., TNF-α, IFN-γ, IL-6, and IL-17A) and increment of the anti-inflammatory cytokine IL-10 in serum, while no significant difference in IL-4 was observed between Con A-treated WT and *fat-1* transgenic mice (Figure 2A). Accordingly, intrahepatic mRNA expression of the pro-inflammatory cytokines was significantly suppressed in *fat-1* transgenic mice compared with those in WT mice upon Con A administration (Figure 2B). Also, the trend of change in the hepatic expression of IL-10 and IL-4 was similar with those in serum between WT and *fat-1* transgenic mice (Figure 2B).

### Activation of T Cells and the Differentiation of Th1 Cells Are Suppressed in *Fat-1* Transgenic Mice Treated with Con A

**Figure 3:**
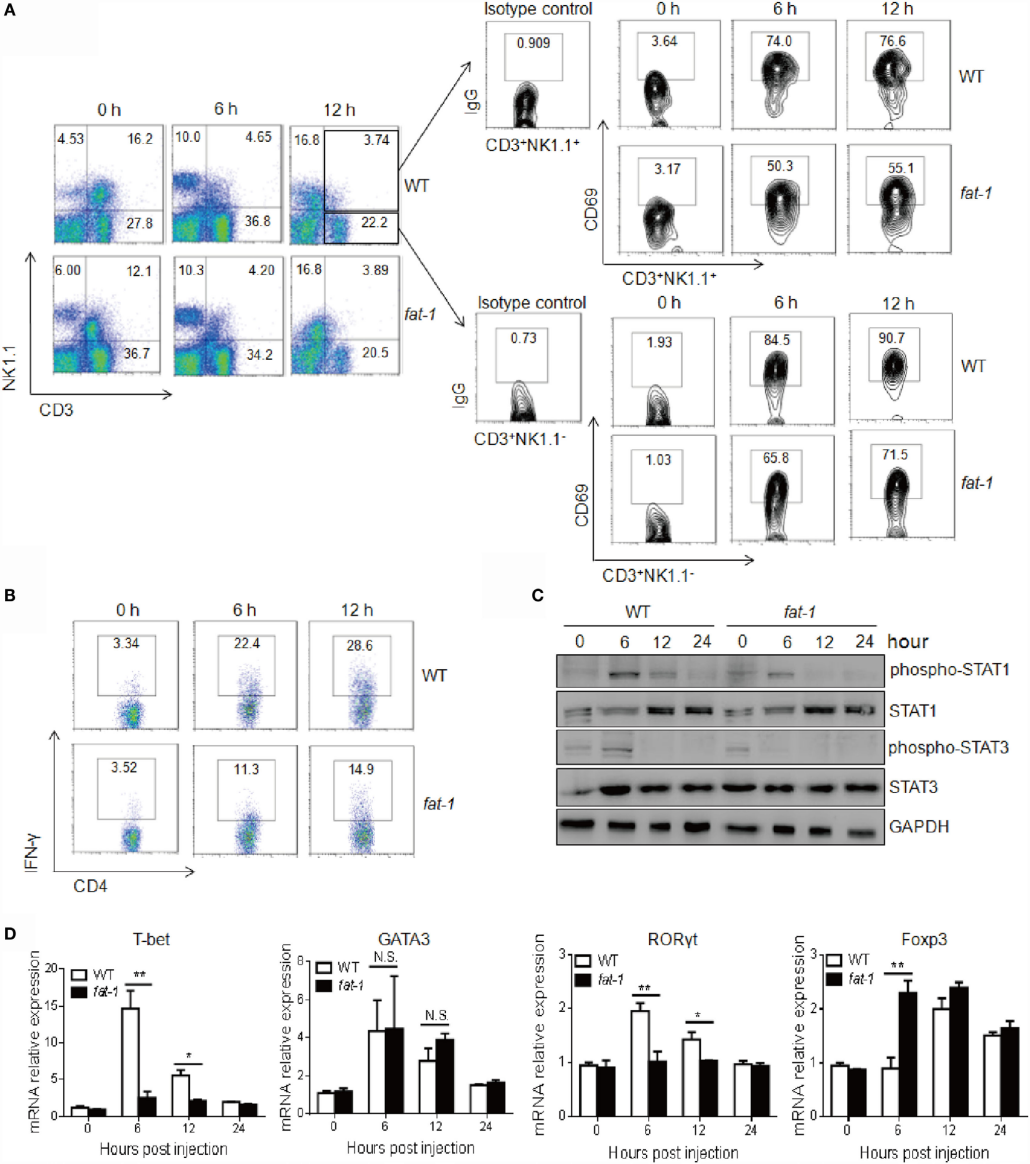
**Endogenous n-3 PUFAs suppress intrahepatic NKT and T cells activation and Th1 cells differentiation upon Con A administration**. Hepatic MNCs were isolated from the liver of WT and *fat-1* transgenic mice (*n* = 5) at indicated time after Con A (15 mg/kg) injection. **(A)** The lymphocyte population and its activation were analyzed by FACS using anti-NK1.1, anti-CD3, and anti-CD69 antibodies. **(B)** Intracellular IFN-γ production of hepatic CD4^+^ T cells was compared by FACS analysis. **(C)** Levels of STAT1 and STAT3 proteins and phospho-STAT proteins in liver lysates from WT and *fat-1* transgenic mice after injection of Con A were analyzed by Western blotting. GAPDH was served as a loading control. **(D)** Relative intrahepatic mRNA expression of T cell transcript factors, including T-bet, GATA3, RORγt, and Foxp3, was measured by quantitative RT-PCR analysis at 0, 6, 12, and 24 h post-Con A administration and expressed as a ratio to GAPDH. *p < 0.05, **p < 0.01, N.S., not significant. One of three independent experiments is shown.

Activation of T cells or NKT cells was also determined in Con A-challenged mice. The frequency of T cells and NKT cells in the liver was decreased in both WT and *fat-1* transgenic mice after Con A injection, and no significant difference was observed between the two groups (Figure 3A). However, hepatic T cells and NKT cells displayed reduced activation in Con A-administrated *fat-1* transgenic mice compared with those in WT counterparts, as indicated by a decreased expression of CD69 (Figure 3A).

Since IFN-γ and CD4^+^ Th1 cells differentiation plays a dominant role in Con A-induced liver damage (4, 7), we therefore evaluated the IFN-γ-producing immune cells in liver. As shown in Figure 3B, Con A-treated *fat-1* transgenic mice showed a marked decrease in the frequency of IFN-γ-producing CD4^+^ T cells in the livers compared to WT controls. Accordingly, liver STAT1 phosphorylation at Tyr701 induced by Con A was much lower in *fat-1* transgenic mice than that in WT littermates (Figure 3C). Meanwhile, reduced STAT3 activation was also observed in the liver of Con A-administrated *fat-1* transgenic mice (Figure 3C), which was possibly due to the reduced IL-6 level in serum of *fat-1* transgenic mice. T-bet is a transcription factor critical to the development of CD4^+^ Th1 cells (22). We demonstrated that T-bet was greatly suppressed in the liver of *fat-1* transgenic mice compared to their WT counterparts, suggesting that endogenous n-3 PUFAs could suppress the differentiation of Th1 cells in Con A-induced hepatitis. By contrast, the Th2 transcript factor GATA3 expression was comparable in these two groups with or without Con A administration. Surprisingly, the Th17 transcription factor RORγt expression was lower, while the Treg transcript factor Foxp3 expression was higher in liver from *fat-1* transgenic mice than those from WT mice after Con A injection (Figure 3D).

### Suppression of the *In vitro* Activation of Hepatic T Cells by n-3 PUFAs

**Figure 4:**
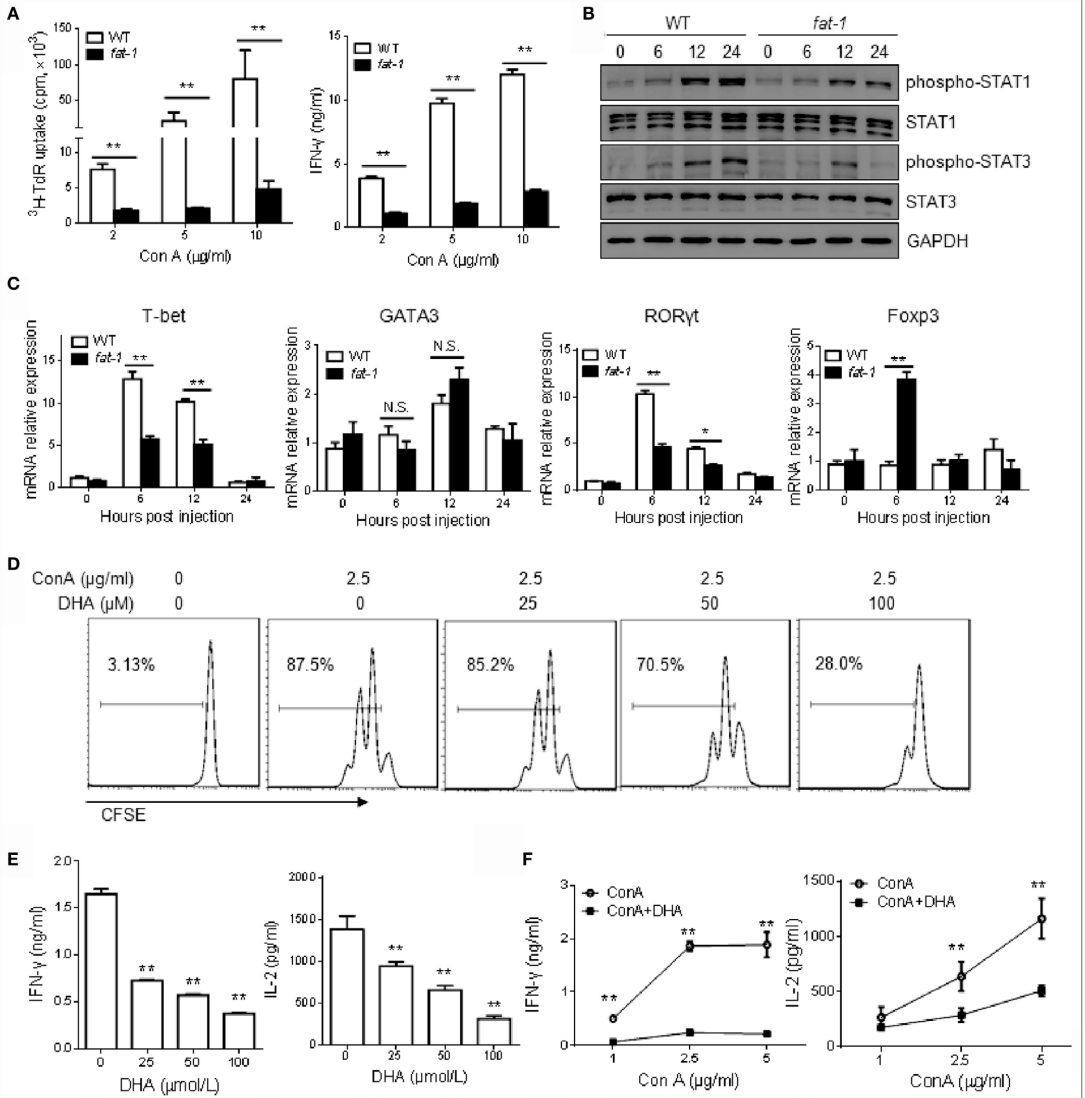
**n-3 PUFAs inhibit Con A-stimulated T cell activation *in vitro***. **(A)** Hepatic MNCs from WT and *fat-1* transgenic mice were stimulated with different concentration of Con A *in vitro*. The proliferation response and cytokine production of T cells were determined by [^3^H] thymidine uptake and ELISA assay, respectively. **p < 0.01. Data are representative of three independent experiments. **(B,C)** Hepatic MNCs were treated with Con A (5 μg/ml) for various time. Cell extracts were then subjected to western blotting analysis with the antibodies against phospho-STAT1, phospho-STAT3, STAT1, and STAT3. One of the three independent experiments is shown **(B)**. Relative mRNA expression of T cell transcript factors, including T-bet, GATA3, RORγt, and Foxp3, was measured by quantitative RT-PCR analysis and expressed as a ratio to GAPDH. *p < 0.05, **p < 0.01, N.S., not significant. One of the three independent experiments is shown **(C)**. **(D,E)** Liver MNCs isolated from WT mice were cultured with Con A (2.5 μg/ml) in the presence of indicated doses of DHA. T cell proliferation was measured by CFSE dilution **(D)**, and cytokine levels were determined by ELISA assays **(E)**. **p < 0.01, compared to the group without DHA. Data are representative of three independent experiments. **(F)** Liver MNCs isolated from WT mice were stimulated with Con A at different concentrations with or without DHA (50 μmol/L) incubation, and the cytokine production was evaluated by ELISA. **p < 0.01, compared to the group without DHA. Data shown represent three independent experiments with similar results.

To determine whether endogenous n-3 PUFAs influence the Con A-induced T cell activation *in vitro*, hepatic MNCs isolated from WT and *fat-1* transgenic mice were stimulated with different concentrations of Con A. As shown in Figure 4A, [^3^H] thymidine uptake and IFN-γ production were reduced in Con A-stimulated liver MNCs from *fat-1* transgenic mice compared to those from WT mice. Moreover, the phosphorylation of STAT1 or STAT3 was much lower in Con A-stimulated liver MNCs derived from *fat-1* transgenic mice than those in WT controls (Figure 4B). Besides, the mRNA expression of T-bet and RORγt was greatly suppressed, whereas Foxp3 mRNA expression was increased in the Con A-treated liver MNCs from *fat-1* transgenic mice compared to those from WT mice. In contrast, the GATA3 expression was comparable in both groups with or without Con A treatment (Figure 4C).

Furthermore, we stimulated liver MNCs from WT mice with Con A in the absence or presence of DHA, which is a form of n-3 PUFAs. DHA was found strongly to inhibit the T cell proliferation (Figure 4D) and cytokines (i.e., IFN-γ and IL-2) production (Figure 4E) in a dose-dependent manner. Likewise, we evaluated the function of DHA on hepatic T cells activation induced by different concentration of Con A. The result also showed that DHA has an inhibitory effect on T cell activation, as indicated by decreased secretion of IFN-γ and IL-2 (Figure 4F).

### n-3 PUFAs Enhance Autophagy Activity in Immune Cells upon Con A Administration

**Figure 5:**
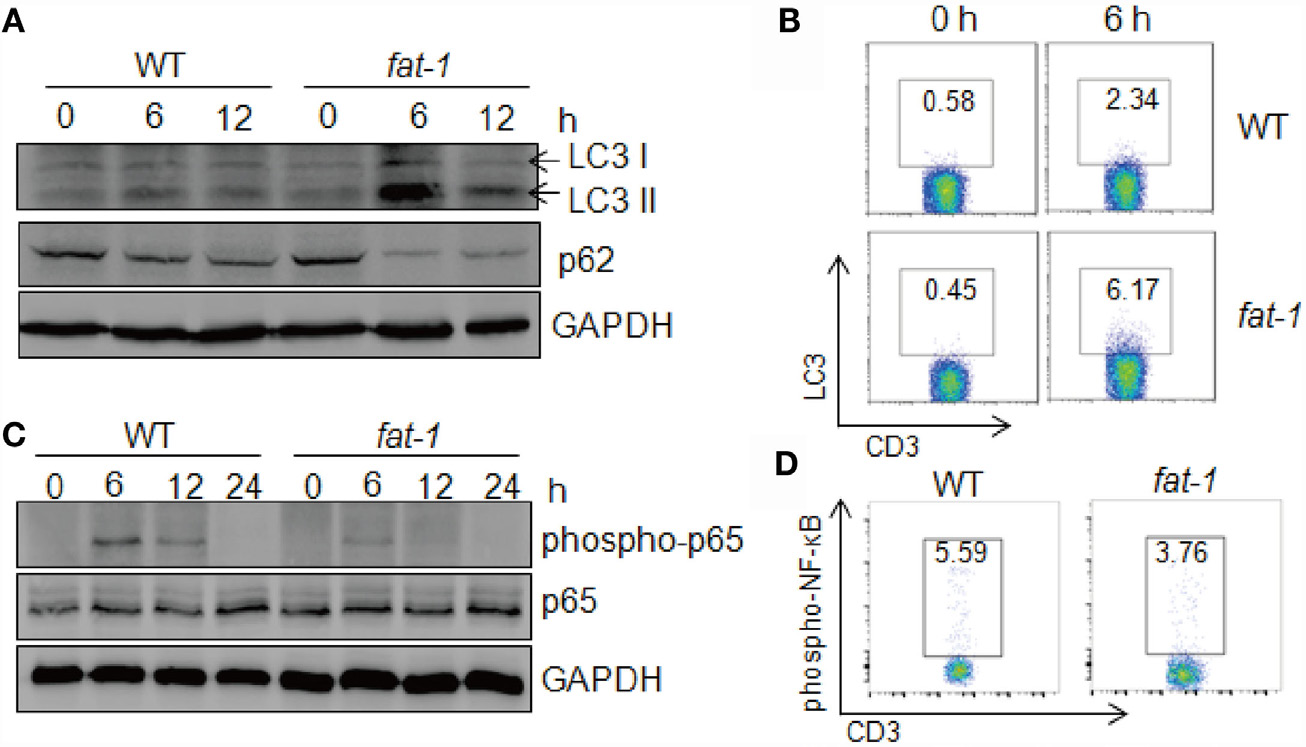
**Endogenous n-3 PUFAs enhance autophagy activity in immune cells upon Con A administration**. **(A)** Autophagy associated protein LC3 and p62 in liver tissues were analyzed by western blotting analysis at indicated time after Con A (15 mg/kg) injection into WT and *fat-1* transgenic mice (*n* = 5). **(B)** The frequency of LC3-producing hepatic T cells was assessed at 6 h after Con A (15 mg/kg) injection by intercellular staining and FACS analysis. **(C,D)** Reduced NF-κB activation in the livers of Con A-administrated *fat-1* transgenic mice (*n* = 5). Levels of phospho-NF-κB p65 and total NF-κB p65 protein in liver lysates from WT and *fat-1* transgenic mice were examined by immunoblotting at indicated time following Con A (15 mg/kg) injection **(C)**. The activity of NF-κB in hepatic T cells from WT and *fat-1* transgenic mice was evaluated at 6 h after Con A (15 mg/kg) injection by FACS analysis **(D)**. Data are representative of three independent experiments.

Autophagy serves as a cellular protective mechanism, helping maintain normal cellular functioning and homeostasis (23). In acute liver injury, autophagy plays a protective role, and autophagic cell death occurs in the failure of adaptation (24). To investigate whether autophagy is associated with the protective effect of n-3 PUFAs in Con A-induced hepatitis, we assessed the expression of proteins related to autophagy in the liver post-Con A injection. The result showed that LC3-II level dramatically increased in liver tissue from Con A-treated *fat-1* transgenic mice compared with that from WT mice (Figure 5A). Also, the expression of p62 was low in *fat-1* transgenic mice as compared with that in WT mice. Indeed, FACS analysis showed a much higher frequency of LC3 positive T cells in the liver of *fat-1* transgenic mice compared to their WT counterparts (Figure 5B). These results suggested that n-3 PUFAs enhanced the autophagy activity in hepatic T cells from mice with Con A treatment. Given the interplay between autophagy process and NF-κB signaling pathway is necessary for the maintenance of cellular homeostasis (25), we examined the NF-κB activity in the liver tissue and hepatic T cell upon Con A stimulation. Reduced phosphorylation of NF-κB p65 was observed in the liver of Con A-treated *fat-1* transgenic mice (Figure 5C). Also, NF-κB activity in hepatic T cells from Con A-injected *fat-1* transgenic mice was lower than those from WT counterparts (Figure 5D).

**Figure 6:**
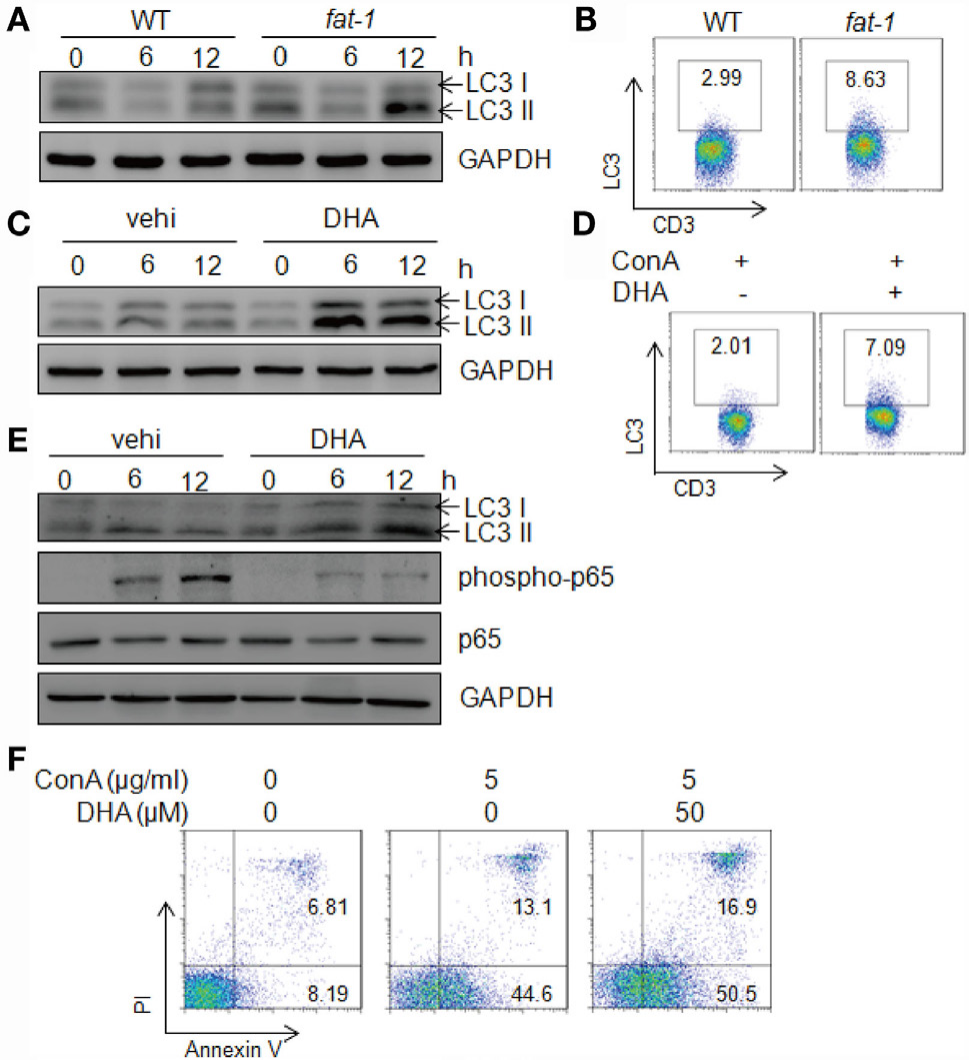
**n-3 PUFAs increase autophagy levels in hepatic T cells stimulated with Con A *in vitro***. **(A)** Autophagy-associated protein LC3 in Con A (5 μg/ml)-stimulated hepatic MNCs from WT and *fat-1* transgenic mice were analyzed by western blotting analysis at indicated time. **(B)** The frequency of LC3-producing hepatic T cells was assessed at 12 h after Con A stimulation by intercellular staining and then FACS analysis. **(C)** Autophagy-associated protein LC3 in Con A (5 μg/ml)-cultured hepatic MNCs in the presence or absence of DHA (50 μmol/L) by western blotting analysis. **(D)** The frequency of LC3-producing hepatic T cells was assessed at 12 h after Con A stimulation with or without DHA treatment by intercellular staining and then FACS analysis. Data shown represent three independent experiments with similar results. **(E)** Autophagy-associated protein LC3 and NF-κB p65 in Con A (5 μg/ml)-stimulated hepatic T cells with or without DHA (50 μmol/L) treatment were analyzed by western blotting analysis at indicated time. Data are representative of three independent experiments. **(F)** Hepatic T cells purified from WT mice were incubated with Con A (5 μg/ml) in the presence or absence of DHA (50 μmol/L) for 24 h. Cells apoptosis were determined by Annexin V/PI staining. Data shown represent three independent experiments with similar results.

Next, we evaluated the autophagy levels in hepatic MNCs stimulated with Con A *in vitro*. Isolated hepatic MNCs and T cells from *fat-1* transgenic mice exhibited higher autophagy activity compared with those from WT mice upon Con A stimulation (Figures 6A,B). Additionally, DHA elevated the expression of LC3 in hepatic MNCs and T cells induced by Con A incubation, as showed by immunoblotting and FACS analysis (Figures 6C,D). To further define whether n-3 PUFAs directly affect autophagy activity in T cells, hepatic T cells were purified from WT mice and stimulated with Con A *in vitro* in the presence or absence of DHA. The result showed that DHA significantly increased autophagy activity and suppressed NF-κB p65 phosphorylation in Con A-stimulated hepatic T cells (Figure 6E). Autophagy has also been associated with the regulation of various cell death pathways, most notably apoptosis (26). Thus, we evaluated the effect of n-3 PUFAs on Con A-induced cell apoptosis in hepatic T cells, and the result showed that DHA increased apoptosis of Con A-activated T cells (Figure 6F). It indicated that n-3 PUFAs limit T cell immune response at least partially by promoting cell apoptosis of activated T cells.

### Inhibition of Autophagy with Chloroquine Abrogates the Protective Function of n-3 PUFAs in Con A-Induced Hepatitis

**Figure 7:**
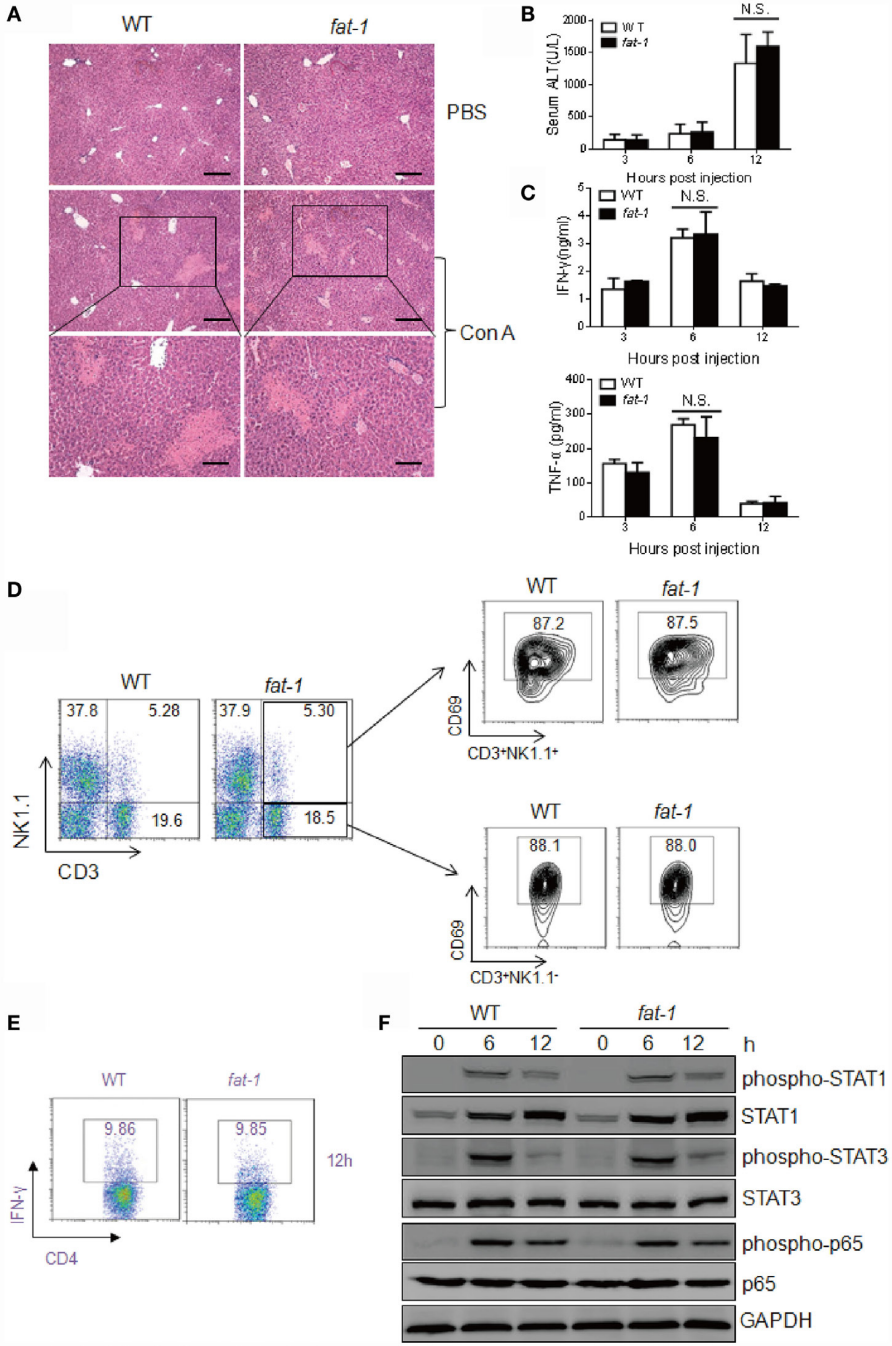
**Chloroquine abolishes the protective function of endogenous n-3 PUFAs in Con A-induced liver injury**. WT and *fat-1* transgenic mice (*n* = 5) received 40 mg/kg body weight of chloroquine by intraperitoneal injection before Con A (15 mg/kg) challenge. **(A–C)** Histology of liver was examined at 24 h following Con A administration. Scale bars = 100 μm. Bottom panels showed the higher-magnification views of the necrotic area. Scale bars = 50 μm **(A)**. Serum ALT **(B)** and cytokine levels **(C)** were evaluated at indicated time. N.S., not significant. One of the three independent experiments is shown. **(D,E)** The percentage of CD69^+^ T cells and NKT cells **(D)** as well as the frequency of IFN-γ-producing CD4^+^ T cells **(E)** were assessed at 12 h after Con A injection by FACS analysis of hepatic MNCs. Data shown represent three independent experiments with similar results. **(F)** Levels of phospho-STAT1, phospho-STAT3, phospho-NF-κB p65, STAT1, STAT3, and NF-κB p65 protein in the liver from Con A-administrated mice with chloroquine pretreatment were analyzed using immunoblotting assay at indicated time. One of the two independent experiments is shown.

Chloroquine inhibits autophagy as it raises the lysosomal pH, thus blocking the fusion between autophagosomes and lysosomes (27). To further study the role of autophagy in the protective function of n-3 PUFAs in Con A-induced liver damage, WT and *fat-1* transgenic mice received 40 mg/kg of chloroquine to block autophagy before Con A administration, followed by histological and serum analysis. The severity of liver damage was comparable between Con A-injected WT and *fat-1* transgenic mice with chloroquine pretreatment (Figure 7A). Additionally, the sera collected from WT and *fat-1* transgenic mice showed no significant difference in the levels of ALT activity (Figure 7B) and pro-inflammatory cytokines (i.e., IFN-γ and TNF-α) (Figure 7C). Analysis of hepatic MNCs showed that the frequency and the activation of NKT cells or T cells were similar between WT and *fat-1* transgenic mice upon Con A challenge with autophagy inhibition (Figure 7D). The percentage of IFN-γ-producing CD4^+^ Th1 cells in *fat-1* transgenic mice was almost the same with those in WT mice (Figure 7E). Moreover, no significant difference was seen between WT and *fat-1* transgenic mice in the phosphorylation of STAT1 and STAT3 as well as the NF-κB activity (Figure 7F). These data suggested that the protective effect of endogenous n-3 PUFAs on Con A-induced hepatitis is dependent on autophagy in regulating T cell activation.

## Discussion

Omega-3 polyunsaturated fatty acids have been reported to attenuate various liver diseases and cancer (15–17, 28). However, the function of n-3 PUFAs in autoimmune hepatitis remains unclear. In the present study, we demonstrate that endogenous production of n-3 PUFAs in *fat-1* transgenic mice reduces the Con A-induced T cell-mediated hepatic injury. Our studies revealed the inhibitory effect of endogenous n-3 PUFAs on Con A-induced T cell activation and CD4^+^ Th1 differentiation. Furthermore, we also provided the evidence that n-3 PUFAs enhanced autophagy activity, resulting in protection from severe liver damage due to the acute overreaction of the immune system.

Previous *in vitro* and *in vivo* studies showed that n-3 PUFAs reduced pro-inflammatory cytokines production secreted from monocytes and macrophages. For example, cell culture studies showed that n-3 PUFAs can suppress the production of TNF-α by MNCs (29) and IL-6 and IL-8 by endothelial cells (9, 30). Schmöcker et al. demonstrated that *fat-1* transgenic mice developed less severe macrophage-mediated liver injury induced by d-GalN/LPS than WT mice, as indicated by decreased plasma TNF-α level, ALT activity, and liver pathology (15). Here, we demonstrated that endogenous n-3 PUFAs also inhibited the pro-inflammatory cytokines produced by activated T cells. These results extended our understanding of the negative regulatory function of n-3 PUFAs on inflammatory responses triggered by various immune cells. It is quite important to notice that the liver macrophages are responsible for the proliferation of CD4^+^ T cells and generation of IFN-γ-producing Th1 cells in Con A-induced hepatitis (31). Therefore, it is possible that modulation of T cell function *in vivo* partially owing to the effect of n-3 PUFAs on liver macrophages, and further investigations are warranted.

Since T cells activation plays a significant role in Con A-induced hepatitis (18, 21), the amelioration of Con A-induced liver injury in *fat-1* transgenic mice may also be related to the selective inhibition of activated T lymphocytes by n-3 PUFAs. We determined that endogenous n-3 PUFAs suppressed the activation of T cells and NKT cells during Con A challenge. On autoimmune hepatitis, CD4^+^ T cells represent the predominant population of T cells infiltrating into the liver, and Th1-like cytokines (e.g., IFN-γ and TNF-α) contribute to hepatic injury (4, 19). It has been noted that n-3 PUFAs supplementation attenuated Th1 cell-mediated delayed type hypersensitivity responses in healthy human volunteers (32). Here, we observed remarkably suppressed polarization of Th1 cells and reduced expression of Th1 transcription factor, T-bet, in liver from Con A-injected *fat-1* transgenic mice. Additionally, we also observed reduced activation of Th17-related STAT3 as well as expression of Th17 transcript factor (RORγt) in liver and decreased IL-17A level in serum from Con A-administrated *fat-1* transgenic mice. A recent study showed that long-term administration of n-3 PUFAs amplified the number of Tregs in the liver, and abundant IL-10 expression in n-3 PUFAs-feed mice might be partially contributing to the generation of hepatic Tregs (33). It is worth noting that the expressions of Treg transcript factor Foxp3 and immunomodulatory cytokine IL-10 were significantly increased in *fat-1* transgenic mice compared to their WT littermates after Con A administration. Our observations support the conclusion that hepatic n-3 PUFAs could influence the differentiation of CD4^+^ T cells into various effector subsets in the liver during immune-mediated hepatitis, and therefore modulate the pathogenesis of autoimmune and chronic hepatitis.

The autophagy process is often used to eliminate damaged or unwanted organelles and also remove intracellular microbial pathogens (23), which play a significant role in the normal liver physiology, as well as in the pathogenesis of liver disease (24, 34). However, the influence of autophagy in the pathogenesis of T cell-mediated hepatitis remains controversial. In the present study, we provided the evidence that autophagy activity is associated with the function of endogenous n-3 PUFAs in modulating the severity of liver injury induced by Con A. NF-κB signaling, which regulates the transcription of critical effector T cell genes, is crucial for T cell activation (35). Autophagy has been described to downregulate NF-κB activity in effector T cells, *via* degrading the adaptor protein Bcl10, which assembles a signaling complex with Carma-1 and the paracaspase Malt1 for NF-κB activation (36). We found that n-3 PUFAs promoted autophagy activity and inhibited NF-κB p65 phosphorylation in T cells. This effect may be associated with the reduced T cell activation in *fat-1* transgenic mice upon Con A administration. Nevertheless, additional studies are required to elucidate the precise mechanism of autophagy in modulating T cell-NF-κB activity during Con A-induced hepatitis.

Importantly, mammalian target of rapamycin (mTOR) has been identified as a crucial regulator of cell metabolism regulating metabolism and plays a critical role in driving T cell differentiation and function (37). Inhibition of mTOR activity by rapamycin promotes T cell anergy even in the presence of costimulatory activation (38). Emerging evidence showed that increased autophagy is linked to mTOR inhibition in senescent cells (39). The previous study demonstrated that n-3 PUFAs rapidly and efficiently suppressed both mTOR complex 1 (mTORC1) and mTORC2 and their downstream signaling (20). Hence, we speculate that hepatic n-3 PUFAs may regulate mTOR-autophagy axis in the liver during immune-mediated hepatitis.

In summary, we demonstrate a role for endogenous n-3 PUFAs in the alleviation of T cell-mediated hepatic injury. Increased T cell autophagy activity induced by n-3 PUFAs restrains Con A-induced T cell activation and inflammatory liver injury. These findings substantiate the concept that n-3 PUFAs have potential clinical application to attenuate immune-mediated liver injury.

## Author Contributions

DZ and ZC designed research. YL, YT, SW, Jing Zhou, Jia Zhou, and XL performed the experiments. YL, Jia Zhou, XB, and DZ analyzed the data. DZ, ZC, and X-YW wrote the manuscript.

## Conflict of Interest Statement

The authors declare that the research was conducted in the absence of any commercial or financial relationships that could be construed as a potential conflict of interest.

## Abbreviations

ALT, alanine aminotransferase; ALA, alpha-linolenic acid; BSA, bovine serum albumin; CFSE, 5-(and-6)-carboxy fluorescein diacetate succinimidyl ester; Con A, Concanavalin A; DHA, docosahexaenoic acid; EPA, eicosapentaenoic acid; H&E, hematoxylin and eosin; MNCs, mononuclear cells; mTOR, mammalian target of rapamycin; n-3 PUFAs, Omega-3 polyunsaturated fatty acids; NF-κB, nuclear factor-κB; PI, propidium iodide; PVDF, polyvinylidene fluoride; WT, wild-type.
